# How do we reach the girls and women who are the hardest to reach? Inequitable opportunities in reproductive and maternal health care services in armed conflict and forced displacement settings in Colombia

**DOI:** 10.1371/journal.pone.0188654

**Published:** 2018-01-18

**Authors:** Juan Carlos Rivillas, Raul Devia Rodriguez, Gloria Song, Andréanne Martel

**Affiliations:** 1 Initiative of Research in Health Services and Systems, School of Public Health, University of Valle, Cali, Colombia; 2 Faculty of Medicine, University of Valle, Cali, Colombia; 3 Postdoctoral Fellowship, Center for Advanced Orthopedic Studies BIDMC, Harvard Medical School, Boston, United States of America; 4 Faculty of Law, University of Ottawa, Ottawa, Canada; 5 Canadian Council for international Co-operation (CCIC), Ottawa, Canada; London School of Economics and Political Science, UNITED KINGDOM

## Abstract

**Objectives:**

This paper assesses inequalities in access to reproductive and maternal health services among females affected by forced displacement and sexual and gender-based violence in conflict settings in Colombia. This was accomplished through the following approaches: first, we assessed the gaps and gradients in three selected reproductive and maternal health care services. Second, we analyzed the patterns of inequalities in reproductive and maternal health care services and changes over time. And finally, we identified challenges and strategies for reaching girls and women who are the hardest to reach in conflict settings, in order to accelerate progress towards universal health coverage and to contribute to meeting the Sustainable Development Goals of good health and well-being and gender equality by 2030.

**Methods:**

Three types of data were required: data about health outcomes (relating to rates of females affected by conflict), information about reproductive and maternal health care services to provide a social dimension to unmask inequalities (unmet needs in family planning, antenatal care and skilled births attendance); and data on the female population. Data sources used include the National Information System for Social Protection, the National Registry of Victims, the National Administrative Department of Statistics, and Demographic Health Survey at three specific time points: 2005, 2010 and 2015. We estimated the slope index of inequality to express absolute inequality (gaps) and the concentration index to expresses relative inequality (gradients), and to understand whether inequality was eliminated over time.

**Results:**

Our findings show that even though absolute health care service-related inequalities dropped over time, relative inequalities worsened or remain unchanged. All summary measures still indicated the existence of inequalities as well as common patterns. Our findings suggest that there is a pattern of marginal exclusion and incremental patterns of inequality in the reproductive and maternal health care service provided to female affected by armed conflict.

**Conclusions:**

Overall, the effects of conflict continue to threaten reproductive and maternal health in Colombia, impeding progress towards the realization of universal health care (UHC) and reinforcing already-existing inequities. Key messages and steps forward include the need to understand the two distinct patterns of inequalities identified in this study in order to prompt improved general policy responses. Addressing unmet needs in reproductive and maternal health requires supporting gender equality and prioritizing the girls and women in regions with the highest rates of victims of armed conflict, with the objective of leaving no girl or woman behind. This analysis represents the first attempt to analyze coverage-related inequality in reproductive and maternal health care services for female affected by armed conflict in Colombia. As the World Health Organization and global health systems leaders call for more inclusive engagement, this approach may serve as the key to shaping people-centred health systems. In this particular case, health care facilities must be located in close proximity to girls and women in conflict and post-conflict settings in order to deliver essential reproductive and maternal health care services. Finally, reducing inequalities in opportunities would not only promote equity, but also drive sustainable development.

## Introduction

Good health for girls and women is essential for sustainable development, and some aspects have substantially improved in past decades [1]. In fact, worldwide priorities in women's health have been changing from a narrow focus on maternal and child health to the broader framework of sexual and reproductive health rights [2]. Nevertheless, there is still much work to be done to put girls and women first.

Gender equality, the empowerment of girls and women, healthy lives and well-being for all at all ages and the reduction of inequalities within countries have been included for first time on the United Nations’ ambitious agenda, and member states have pledged to tackle the Sustainable Development Goals (SDGs) [3]. Detailed targets by 2030 include: goals 3.7 and 5.6 to ensure universal access to sexual and reproductive health-care services and rights, including family planning, information and education, and the integration of reproductive health into national strategies and programmes; goals 5.1 and 5.2 to eliminate all forms of discrimination against all women and girls everywhere; and goal 10.2 to empower and promote the social, economic and political inclusion of all, irrespective of age, sex, disability, race, ethnicity, origin, religion or economic or other status [4].

Millions of people around the world are caught in the vicious spiral of violent conflict and poor health. Recently, conflicts have dramatically escalated and countries such as Yemen, Syria and South Sudan have become some of the worst humanitarian crises in the world. Large gender gaps exist in countries where women suffer social exclusion and lack of opportunities because of armed conflict and forced displacement. Within the complex and varied settings, access to services such as family planning, antenatal care, abortion, skilled births attendance and emergency obstetric care can too easily be compromised or denied [3, 4, 5]. As such, these health-related SDG targets require a pro-equity approach that promotes accelerated progress for the poorest and most disadvantaged communities within countries [5]. This also means reaching the poorest and most vulnerable girls and women in conflict and post-conflict contexts, and putting them first when designing programs and agenda to tackle inequalities as the Global Strategy for Women's, Children's and Adolescents' Health urgently calls for action.

Colombia just signed a historic peace deal with the Revolutionary Armed Forces of Colombia (FARC (for its acronym in Spanish) to end more than 50 years of conflict. Despite this important step forward to end the armed conflict, these conflict and post-conflict settings remain complex and there is still much to do. As of April 2017, the National Registry of Victims (RUV) estimates that there are over 8.1 million victims of armed conflict in Colombia [6]. The estimated population of Colombia is 45 million [7], which means Colombian victims represent 18% of the Colombian population. The majority of victims (4.5million) were females affected by forced displacement and sexual and gender-based violence, and were mostly female adolescents, single mothers or widowed with children affected by the war. At least 40% of the victims were women below the age of 29; approximately 10% were girls and young women between 10–19 years old; about 40% were adult women between 30–59 years old; 13% were older women above the age of 65; and 4% were octogenarian’s women over 80 years old. Yet, some researchers [7, 8] suggest this data systematically misrepresents reality, and does not accurately reflect the true number of victims of conflict in Colombia. Moreover, we need to consider the challenges posed by ending the armed conflict and signing a peace agreement with the other guerrilla groups and rebels.

As a result, in 2012, the latest National Health Plan 2012–2021 [9] recognized victims of armed conflict as a vulnerable target group, and called for the strengthening of efforts to tackle inequalities. In particular, it emphasized the need to address the lack of access to basic health care and the delivery of health care benefit packages in reproductive and maternal health, mainly to girls and women in poor, conflict and post-conflict communities. Data from the most recent 2015 Demographic Health Survey (DHS) indicates that family planning (91.7%), antenatal care (97.5%) and skilled birth attendance (97.5%) are closer to meet universal coverage in reproductive and maternal health services [10]. Although most women are covered, evidence suggests that the gap between unmet and met needs in family planning and antenatal care is especially high among groups such as poor and rural women, female adolescents [9, 10], migrant women from Venezuela, and female victims of conflict. Moreover, forced displacement and the slow implementation of protection mechanisms have increased their vulnerability. In fact, many of the opportunities provided by health care services continue to be missed by adolescents and pregnant women. For instance, they may not have suitable contraceptive methods, or they may receive only one antenatal visit instead of four [11].

Evidence that was produced before this study identified the impact of conflicts on access to health care and its gendered implications particularly in relation to sexual and reproductive health services. While more men are injured or die from violence during wars due to the enrollment-armed groups [12, 13], women are particularly vulnerable and face increased risks of sexual and gender-based violence (SGBV) and forced displacement [14, 15, 16]. Due to their reproductive roles, women typically have a heightened vulnerability to ill health and a greater need for health services. These vulnerabilities increase in conflict situations, and as a result, structural inequalities rooted in gender norms impact women’s health [13]. For instance, studies have identified that during conflicts, girls and women experience poor access to essential sexual and reproductive health services [17], high rates of unsafe abortions [13, 14], and increases in maternal mortality [12]. In summary, the evidence strongly suggests girls and women in conflict settings are more likely to face social inequalities accessing sexual, reproductive and maternal health care, and these inequalities may worsen or broaden over time.

However, there is a growing recognition that inequalities are multidimensional (on the basis of age, sex, disability, race, ethnicity, origin, religion, health care, economic or other status), reflected in the recent significant increase analyses and publications [2, 5, 18]. This has prompted action on many different fronts, arguing the importance of capturing different dimensions of inequality. This also has relevance on a practical level, as different dimensions of inequality often require different social interventions [19]. From this perspective, monitoring inequalities is fundamental to an equitable and progressive realization of universal health coverage. Disaggregated data is pivotal to identifying where and why inequalities exist, and ensuring that policies, programmes and practices are successful in reaching the most vulnerable and delivering affordable reproductive and maternal health services. In this particular case, this means reaching girls and women who are the hardest to reach in conflict and post-conflict settings.

Many researchers have documented inequalities in reproductive and maternal health care services on a global level [2, 20, 21] as well as at the national level and cross-countries [9, 10, 22, 23]. However, further research is needed to understand the patterns of inequalities in reproductive and maternal health coverage among female affected forced displacement and sexual and gender-based violence in conflict settings in Colombia. We hypothesize that despite the progressive realization of the universal access to reproductive and maternal health services in Colombia, the female affected by conflict has less opportunities in the delivery of these services.

This paper assesses inequalities in access to reproductive and maternal health care services among females affected by forced displacement and sexual and gender-based violence in conflict settings in Colombia. This was accomplished as follows: we first assessed the gaps and gradients in three selected reproductive and maternal health care services. Second, we analyzed the patterns of inequalities and changes over time. And finally, we identified challenges and strategies for reaching women who are the hardest to reach in conflict settings, in order to accelerate progress towards universal health care (UHC) and to contribute to meeting the SDGs by 2030.

## Materials and methods

### Study design and data sources

This study was a cross-sectional analysis. We used secondary data from 2005, 2010 and 2015 from the registries and cross-sectional nationally representative surveys with data disaggregated at the sub-national level, available on the Information System for Social Protection (SISPRO) [23]. This platform offers access to registry-based databases such as the Civil Registration Vital Statistics (CRVS), social protection, vulnerable populations, and national population-based surveys. SISPRO is provided by the Ministry of Health and Social Protection (MoHSP) of Colombia and we aimed to take advantage of these new developments.

Based on a new resource, the State of Inequality: Reproductive, Maternal, Newborn and Child Health Report [2], we used three types of data to quantify inequalities: i) health outcomes (relating to rates of female affected by conflict); ii) information about access to reproductive and maternal health care services to provide a social dimension that allows ranking the population from worst-off to better off throughout the levels of access, and iii) data on population size (number of women and girls in whole population).

Our explanatory variable was the number of female affected by armed conflict expressed for 1000 women per year. We estimated the numbers in each geographic unit using the number of females of all ages affected by conflict from the National Registry of Victims (RUV for its acronyms in Spanish) [6], and female population data from National Administrative Department of Statistics (DANE) [24]. RUV routinely registers information specific types of victimization in the context of armed conflict in Colombia and allows disaggregation of data at sub-national level. Data was available for 96.1% of registries (2005 = 258.714; 2010 = 131.345; 2015 = 96.422) and 3.9% of the data was missing (Not Reported). DANE provides annual demographic projections of population data at the sub-national level.

For this study, unmet needs in three selected reproductive and maternal health care services were examined as social dimensions to unmask inequalities. Three indicators from the Demographic and Health Survey (DHS) were studied: i) unmet needs in family planning, ii) unmet antenatal care, and iii) unmet needs in births attended by skilled health personnel. DHS is a large-scale, nationally representative household survey that is conducted routinely every five years. These indicators represent a common source of social exclusion, discrimination and lack of opportunities, reflective of progress (or lack of progress) in attaining equity in access to reproductive and maternal health. These indicators are highly recommended by the State of Inequality in Reproductive, Maternal, Newborn and Child Health RMNCH report from the World Health Organization (WHO).

Further details about the selected study outcomes and dimensions of inequality are listed in Table 1.

**Table 1:** Selected health study outcomes and dimensions of inequality.

Item	Category	Indicator	Data sources	Availability
i	Study outcome	Number of female victims of armed conflict per 1 000 women	National Registry of Victims RUV	2005, 2010, 2015
ii	Dimension of inequality	Proportion of women with unmet needs in contra conceptive methods (%)	Demographic and Health Survey (DHS)	2005, 2010, 2015
iii	Dimension of inequality	Proportion of women with unmet needs in antenatal care control (%)	Demographic and Health Survey (DHS)	2005, 2010, 2015
vi	Dimension of inequality	Proportion of women with unmet needs in births attended by skilled health personnel (%)	Demographic and Health Survey (DHS)	2005, 2010, 2015

The analyses relied on data from three different sources, which were combined into panel data disaggregated at the sub-national level (32 departments and Bogota, D.C) for monitoring inequalities in reproductive and maternal health services: RUV, DHS and DANE. We used three specific points in the time: 2005, 2010 and 2015. This panel of data, large data sets including raw data, can be accessed using the public and open Institutional Digital Repository (RID in Spanish) of the MoHSP in the following link: https://www.minsalud.gov.co/sites/rid/Lists/BibliotecaDigital/RIDE/VS/ED/GCFI/PA seguimiento víctimas.zip.

The population of females affected by armed conflict were categorized into deciles (ten subgroups) and quintiles (five subgroups). This segmentation of our study was done in order to simplify the interpretation of the inequalities and to easily communicate the information to be understood by non-technical audiences. This approach has been applied widely by experts monitoring inequalities in health studies [2, 5, 19, 25]. The use of deciles/quintiles to conceptualize health care service-related inequalities helps to address the issue of masked disparities [22]. In our analysis, the three selected indicators had a negative inclination, meaning that more is worse. Therefore, the information must be interpreted as follows: the D1 and Q1 represent the subgroup of departments in the less opportunities or worst situation (higher unmet needs), while the D10 and Q5 represents those in the most opportunities or better situation (much lower unmet needs).

### Measures of inequalities

The rate of females affected by armed conflict was analyzed by examining unmet needs in family planning, antenatal care and skilled births attendance. We calculated two inequality indices to assess absolute and relative inequalities in reproductive and maternal health care services among female victims of armed conflict within the country. These two indices account for the entire distribution of the sample by unmet needs. First, we estimated the slope index of inequality (SII) [2, 18, 25] by regressing the rates of females affected by conflict on the corresponding values for quartiles of unmet needs, in order to assess the absolute gaps for ordinal groups. The line represents the slope of inequality or the gap. Higher numerical values indicate more pronounced inequalities. Negative values indicate that inequality is pro-poor (affecting the departments with less opportunities), while positive values indicate that inequality affects the better-off departments (most opportunities) throughout the social scale. The ridit corresponds to the cumulative frequency of half of that group, listed in order by unmet indicator.

We then estimated the concentration index (CIX) [2, 18, 25] by fitting a Lorenz concentration curve equation to the observed cumulative relative distributions of the whole population, ranked by unmet needs and the rates of females affected by conflict across subnational units. The curve of Lorenz is drawn by connecting the dots. It is expressed on a scale from –1 to +1, on which there is no inequality if the concentration index is 0. The concentration curve lies below the 45° diagonal line from the bottom left corner to the top right. If the indicator is concentrated among the better-off groups, the concentration curve lies above the line of equality. Conversely, if indicator is concentrated among the worst-off groups, then it is under the line of equality. There is no inequality if the concentration curve lies on the line of equality. The slope index of inequality expresses absolute inequality (which expresses the gap or differences between the subgroup with less and most opportunities), whereas the concentration index expresses relative inequality and measures how far the distribution of the access indicator is from a totally equity distribution (gradient). The slope index of inequality is used to study gaps, while concentration index indicates whether inequality is being eliminated over time.

Finally, inequalities have been shown in an equiplot, through a graph displaying a sequence of dots on a line, with each dot representing a reproductive and maternal health care service. Dots that are farther to the right represents a higher rate of females affected by armed conflict. Each dot represents one quintile of unmet needs, from the less severe unmet needs (in this study light blue dot) to the worst unmet needs (in this study black dot). This graph as a result ranks the whole population by unmet needs and population weight by quintile. The data were exported, saved and customized in different formats by the authors with help of a graphic designer.

### Statistical analysis

We completed all statistical analyses using Epidat 4.2 using the module for measuring inequalities. Epidat is an open and publicly accessible source provided by the Pan-American Health Organization (PAHO) and the World Health Organization, and can be accessed at the following link: http://www.sergas.es/Saude-publica/EPIDAT-4-1?idioma=es&print=1

### Study limitations

As with any cross- and sub-national analyses, our study has significant limitations. First, it should be noted that our study assessed the degree of inequality using only three single indicators in reproductive and maternal health care services. However, there are other relevant dimensions of inequality that represent a common source of social exclusion and reflect lack of opportunities. Second, the selected reproductive and maternal health care services only consist of one smaller aspect of a larger reproductive and maternal health framework. And third, qualitative participative approaches such as case studies collecting perceptions of inequalities held by women themselves is needed to fully understand the existence of inequalities.

### Ethics approval

All analyses were based on publicly available data from SISPRO at the Ministry of Health and Social Protection. Ethical approval was not required for this study because the authors did not collect new data and the study relied solely on existing and publically available secondary data. Thus, ethical clearance is the responsibility of the institution that administered the data.

## Results

**Fig 1:**
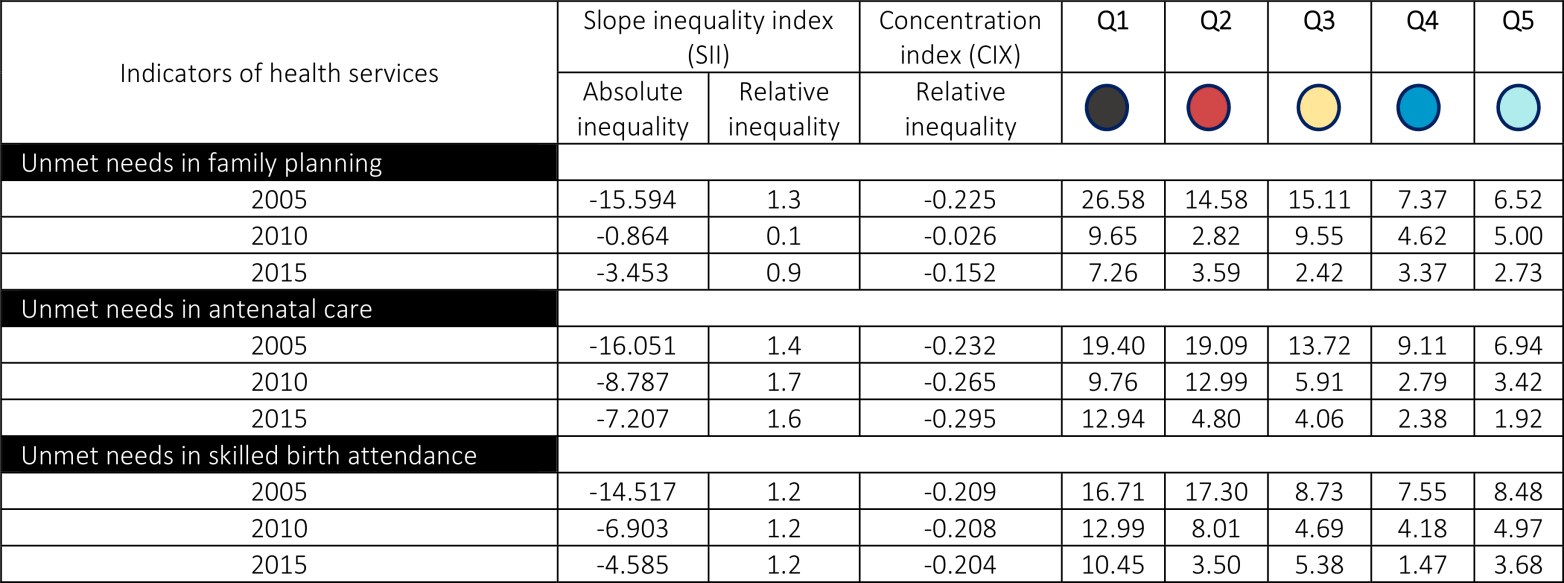
Absolute and relative inequalities in reproductive and maternal health care services among females affected by armed conflict in Colombia in 2005, 2010 and 2015 by quintile.

The slope inequality index and concentration index values for the three indicators are shown in Fig 1. These values are shown along the relative inequality index (RII) to provide an idea of the gaps and gradients in terms of unmet needs in reproductive and maternal health for females affected by armed conflict. In 2005, the magnitude of inequality in the three indicators had a pro-poor inclination. However, the inequality changed substantially between 2005 and 2015, with absolute inequality falling from -15.5 to -3.4 for unmet needs in family planning and from -14.5 to -4.5 for unmet needs in skilled birth attendance. Similar patterns were observed for the relative inequality, which fell from 1.3 to 0.9 for unmet needs in family planning, but increased over that period from 1.4 to 1.6 for unmet needs in antenatal care, despite the fact that the absolute inequality had decreased. On other hand, relative inequality has remained stagnate for unmet needs in births attendance for the entire period that was assessed (1.2).

**Fig 2:**
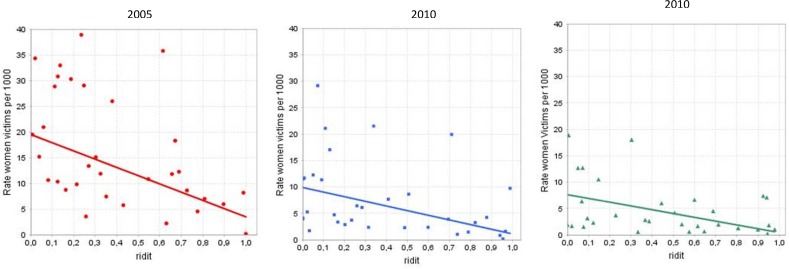
The slope index of inequality for unmet antenatal care among female affected by armed conflict in Colombia, 2005, 2010, 2015 by deciles.

Absolute inequality is captured in the Fig 2 and presents the slope index of inequality for unmet antenatal care among women victims in 2005, 2010, 2015 by deciles. In 2005, there were pronounced inequalities between the worst-off and better-off subgroups (lower unmet needs compared to female subgroups with higher unmet needs). The slope index of inequality were negative over time, indicating pro-poor inequalities, which means higher rates of females affected by forced displacement and sexual and gender-based violence in conflict settings and higher unmet needs at the time. However, inequalities in all health services were halved by 2010, and the gaps dropped slightly between 2010 and 2015 for family planning and skilled birth attendance. Conversely, absolute inequality increased for antenatal care during last ten years-period. In sum, the plot demonstrates that the gap has fallen as the slope flattened over the period assessed.

**Fig 3:**
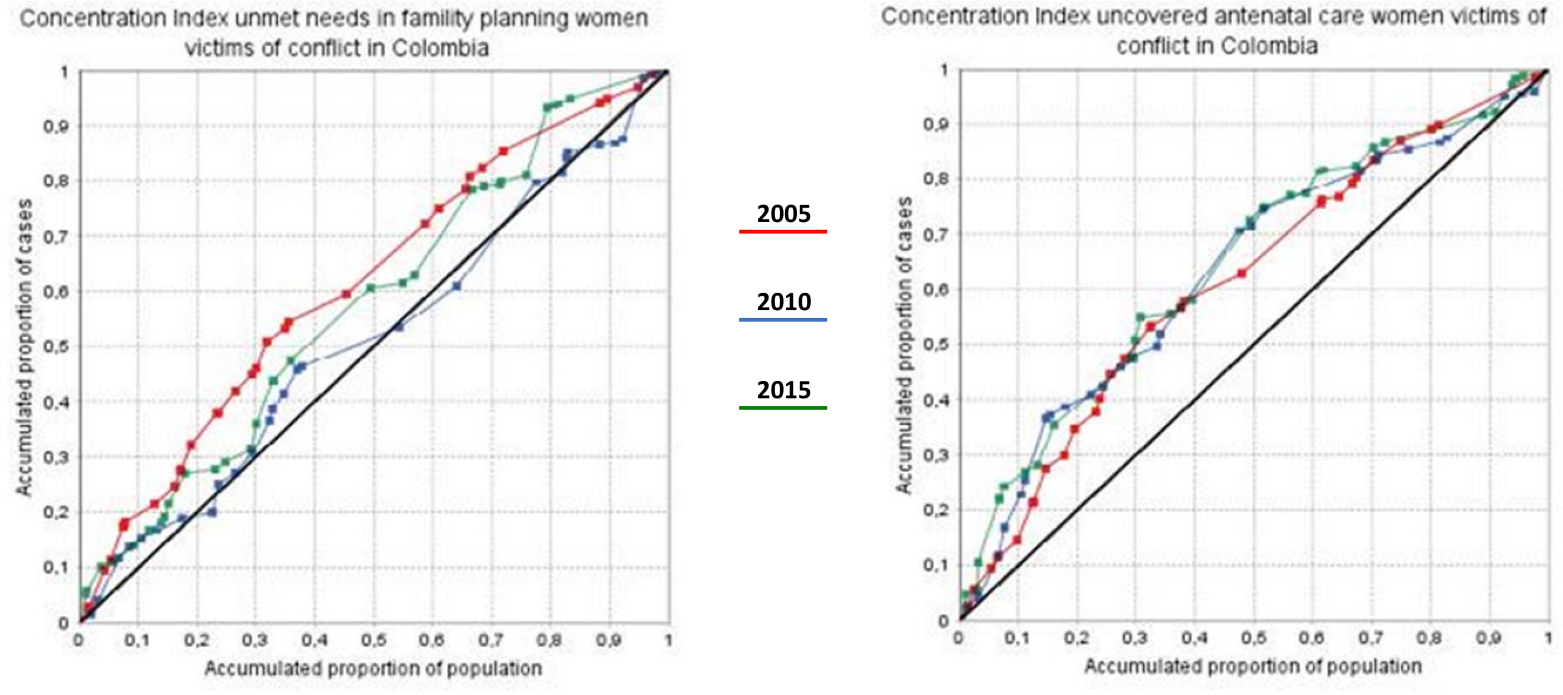
Concentration index in unmet needs in family planning and antenatal care among female affected by armed conflict in Colombia, 2005, 2010, 2015 by deciles.

The changes in relative inequalities (CIX) across the unmet needs in the three selected reproductive and maternal health care services are shown in Fig 3. In 2005, roughly 52% of all females affected by conflict in the country remained disproportionately concentrated in the 32% of the departments with higher unmet needs in family planning. This means that the half of female victims had not opportunities meeting their family planning needs in 2005. Nevertless, this gradient dropped approximately 20% by 2015, which means around 12% female still having problems accessing family planning in conflict settings. Conversely, this gradient remained invariant for unmet antenatal care during the period assessed. Overall, in 2005, around 58% of females affected by armed conflict were concentrated in 38% of the departments with the most unmet needs in antenatal care. The gradient stayed disproportionally concentrated in 2015.

**Fig 4:**
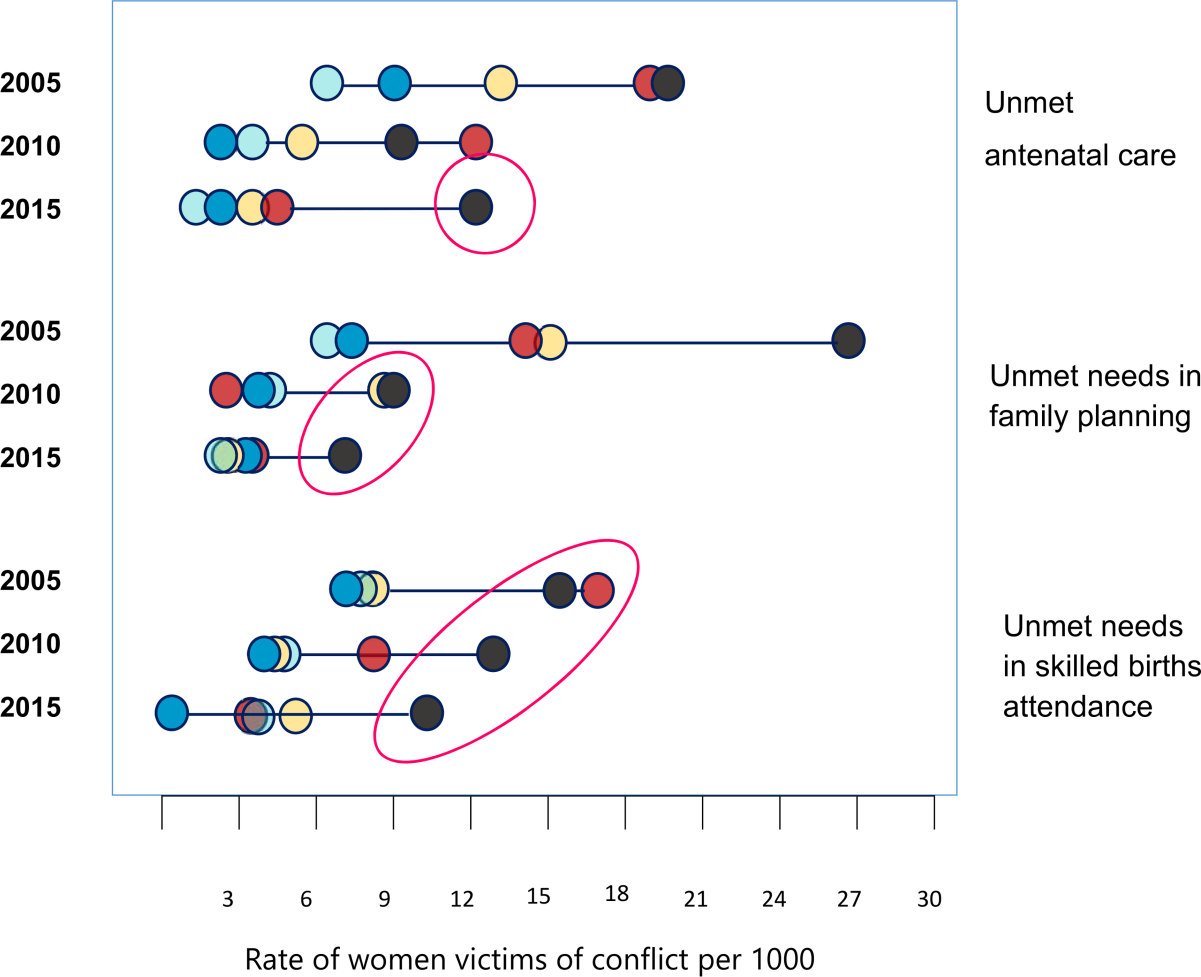
Patterns of inequalities in reproductive and maternal health care services among female affected by armed conflict in Colombia, in 2005, 2010 and 2015 by quintiles.

The patterns of inequalities in access to health care service for female affected by armed conflict, disaggregated by unmet needs in reproductive and maternal health services and changes over time are shown in Fig 4. Despite significant reductions in inequality for the period assessed, the subgroup of departments with higher victims and unmet needs still lag behind. The most inequity health care service was family planning in 2005. In other hand, a reduction in the gap for unmet antenatal care was observed for all quintile subgroups except in Quintile 1 (Vaupés, Chocó, Guainía, Nariño, Putumayo, Vichada, Huila) where the number of female affected by conflict increased. Additionally, inequality in unmet antenatal care seems to have stayed the same over time as women victims rates remained the same in 2015 compared to the 2010. Similar patterns were observed for unmet needs in skilled birth attendance, but with quintiles 3 (Magdalena, Cesar, Cundinamarca, Quindío, and Bogota). Inequalities for unmet needs in family planning were almost eliminated, compared to the other two health care services. On the other hand, quintile 3 reached most equitable levels, but quintile 1 has not followed this reduction as the same pace.

## Discussion

This analysis represents the first attempt to analyze inequalities in reproductive and maternal health care services for female affected by forced displacement and sexual and gender-based violence in conflict settings in Colombia. Our findings show that even if absolute inequalities can drop over time, relative inequality can still worsen or remain unchanged. Based on the results showing significant reductions of relative inequalities in family planning over time, family planning was found to be the most equitable health care service among females in conflict settings in Colombia. Unfortunately, the situation was not the same for antenatal care and skilled birth attendance, where a gap is still present and seems to remain unchanged over time, despite closing across the quintiles of unmet needs. This indicates that inequality has maintained the same size over time.

Another key finding is that all summary measures indicated the existence of two common patterns of inequality. First, a review of the disaggregated data indicates that quintile 1 (which represents the 20% of departments with higher unmet needs) has had significant improvements in reducing the gap compared to other quintiles, but it is still experiencing social exclusion in assessed reproductive and maternal health services over time. In other words, our findings suggest a pattern of marginalization for 20% of the departments with more females affected by conflict and less access to reproductive and maternal health care services as well. This is also known as top inequality, which is represented by the red circle. While, 40% of the population (quintile 1 and 2) had much higher unmet needs for birth attendance and unmet antenatal care in 2005.

Overall, the proportion of unmet needs of births attended by skilled health personnel was reduced over the 10-year period in the majority of the departments. It worth mentioning that the pace of reduction was faster mainly among quintile 1 and 2, the departments with higher unmet needs and female affected by armed conflict, and quintile 5, the departments with lower unmet needs and armed conflict where inequality almost disappeared. However, considering the social exclusion and the pace of the change, this study reports a favourable situation: most departments (87%) reported coverage of over 80% in the quintile with higher unmet needs and rate of females affected by armed conflict.

The variation in the proportion of unmet needs in family planning was larger among females affected by armed conflict in 2005 across quintiles. All departments improved on average with increases, mainly those with higher proportion of females affected by armed conflict; in turn they reported a significant absolute reduction in unmet needs for this service of at least 7.5 to 26.7 percent points between 2005 and 2015. When considering the pace of reduction in inequality, the change was faster and there were improved and increased access to females for this service. As such, family planning may represent the most equitable reproductive and maternal health care service in Colombia, based on these findings. Although inequality is still present in the quintile of females most affected by armed conflict and there are higher unmet needs for this service, the difference in other subgroups of females is significantly smaller.

Regarding unmet needs in antenatal care, the pace of reduction of inequalities tended to be faster among all quintiles except in quintile 1. In other words, the situation improved for most of departments, and as such inequality has fallen except for the quintile of females most disadvantaged who experienced a higher level of social exclusion. It is worth noting that despite the fact that this inequality was reduced within quintiles 2–5, the magnitude of inequality seems to be the same over time.

Overall the country realized improvements in the three assessed reproductive and maternal health care services, with substantially faster reductions among the females affected by armed conflict with less access in the reproductive and maternal health care services. However, reductions in inequality were also more pronounced in quintile 2 over time.

Second, a small incremental pattern was observed only in unmet antenatal care and unmet needs in family planning in 2005 (the most inequitable year). This pattern is indicated by a linear gradient of female victim’s rates that increases across quintiles in the equiplot. Unfortunately, trends indicate slower progress in antenatal care. Conversely, unmet needs in family planning dramatically decreased by 2015, but there was still some marginal exclusion faced by quintile 1.

In this way, the observed marginal exclusion calls for a targeted approach focusing on departments with higher proportions of female victims. An incremental inequality requires an approach that combines population-wide and targeted health care services, mainly in departments such as Choco, Vaupes, Guainía and La Guajira, departments in which inequality seems to be rising.

In summary, the effects of armed conflict continue to threaten reproductive and maternal health in Colombia, which in turn can easily compromise the realization of universal health coverage UHC while also reinforcing inequities. It worth mentioning that although small inequalities remain in the three assessed health care services, failure to provide reproductive and maternal health care services to females affected by forced displacement and sexual and gender-based violence in conflict settings undermines the goal of universal health coverage within the country. In fact, our results can be contrasted with findings of Barros and colleagues [20]; Restrepo-Mendez and colleagues [21], and Hosseinpoor and colleagues [22], which showed evidence of persistent inequalities in family planning, antenatal care and skilled birth attendance using data on Latin American and Caribbean LAC countries. According to Kruk [26], we urgently need to address the needs of the most marginalized and vulnerable females in complex contexts, and as such, health facilities require greater investment in the quality, efficiency, quantity and innovations for reproductive and maternal health services.

## Conclusions

Considering that the peace agreement will hopefully play a pivotal role in reducing the rates of female victimization in armed conflict, we identified two action areas which may help Colombia to move forward and meet the reproductive and maternal health targets of the SDGs by 2030. First, the identified patterns of inequality in this study prompt different policy responses and different innovative approaches delivering reproductive and maternal health care services. To achieve this, putting the most vulnerable and marginalized female at the top of development priorities is key. This requires differential equity and gendered equity-approaches in the conflict and post-conflict context. In others words, we must find new ways to improve the delivery of health care services such as personalized health benefit packages and ensuring health facilities are located in closer proximity to female in conflict settings. Secondly, the World Health Organization calls for more inclusive engagement to shape people-centred health systems as the key to achieving UHC. In particular, it is essential to address the intersection of multiple vulnerabilities (Afro-Colombian, Indigenous, those with low literacy levels) in order to bringing them to acceptable levels of social opportunities. That is to say, It is critical that the most vulnerable females should be considered first, not last, in the design of health care service programs.

## Supporting information

S1 Fig Panel of data. https://www.minsalud.gov.co/sites/rid/Lists/BibliotecaDigital/RIDE/VS/ED/GCFI/PA%20seguimiento%20v%C3%ADctimas.zip.
(XLSX)
